# Tailoring Anticoagulant Therapy: Managing Interactions Between Carbamazepine and Direct Oral Anticoagulants (DOACs)

**DOI:** 10.7759/cureus.93905

**Published:** 2025-10-05

**Authors:** Tajuddin H Mohammed, Pankaj K Singh, Simon-Turriate Juan Pedro, Erika Giordano, Benjamin Young

**Affiliations:** 1 Internal Medicine, East Surrey Hospital, Surrey and Sussex Healthcare NHS Trust, Redhill, GBR; 2 Geriatrics, East Surrey Hospital, Surrey and Sussex Healthcare NHS Trust, Redhill, GBR

**Keywords:** carbamazepine (cbz), clinical haematology, direct oral anticoagulants (doacs), drug interaction, edoxaban, general practice (general medicine), internal medicine (general medicine), neck of femur fracture

## Abstract

Standard prophylaxis after hip surgery typically involves parenteral anticoagulation. However, this approach may not be feasible for patients with significant comorbidities or barriers to injectable treatments. Oral anticoagulants can be considered, but vitamin K antagonists present compliance concerns, and many direct oral anticoagulants (DOACs) are limited by drug interactions.

A 61-year-old male care home resident with multiple comorbidities, including hypoxic brain injury-related seizures and a severe needle phobia, underwent right hip hemiarthroplasty after sustaining a femoral neck fracture. Parenteral thromboprophylaxis was not a viable option due to the needle phobia, and vitamin K antagonists were excluded because of adherence concerns. The patient's regular use of carbamazepine, a strong enzyme inducer, further complicated anticoagulant selection due to its potential to reduce DOAC effectiveness. After a multidisciplinary review, edoxaban was chosen as the most suitable option.

The patient was managed with a daily regimen of edoxaban 60 mg, with therapeutic drug levels confirmed through monitoring. He remained clinically stable without thrombotic or bleeding complications.

This case highlights the importance of an individualised approach to anticoagulation in complex patients, where both clinical context and drug interactions must be carefully considered and balanced. Edoxaban may represent a feasible strategy in patients receiving carbamazepine, provided that treatment is supported by plasma level monitoring and close follow-up.

## Introduction

There are clear guidelines for thromboprophylaxis following major surgery [1] and extended periods of bed rest, but in rare cases, significant therapeutic challenges can occur. Complex comorbidities, behavioural issues, and polypharmacy may necessitate personalised approaches instead of standard prophylaxis.

We report the case of a 61-year-old man with a history of hypoxic brain injury and seizure disorder on carbamazepine, who sustained a right femoral neck fracture requiring hemiarthroplasty. This patient had a severe needle phobia, and the well-known drug interactions between carbamazepine and commonly used direct oral anticoagulants (DOACs) complicated thromboprophylaxis decisions [2]. Carbamazepine's potent induction of hepatic enzymes reduces the effectiveness of many DOACs, while his needle phobia posed the greatest challenge to administering enoxaparin. A review by Galgani et al. highlighted the lack of definitive guidance in such situations, making this a challenging case where anticoagulation could not be avoided despite clear limitations [3].

A literature review, including the study by Di Gennaro et al., indicated that edoxaban might be a better oral option, as it is less affected by enzyme induction and has a more predictable anticoagulant effect in this context [4]. This case illustrates the management of anticoagulation in medically complex patients where enoxaparin is not feasible. It also highlights the emerging role of edoxaban, a DOAC less influenced by enzyme induction, as a promising option supported by therapeutic drug monitoring. Our experience offers valuable clinical insights into personalised anticoagulant therapy when standard options are contraindicated or unsuitable due to pharmacological and behavioural considerations.

## Case presentation

A 61-year-old male patient, resident of a care home and without any next of kin, who also lacked mental capacity, was admitted following a fall. On evaluation, he was diagnosed with a right-sided neck of femur fracture. His past medical history included hypoxic brain injury resulting in seizures, diagnosed mental illness, and a very severe needle phobia. The patient's renal function is normal, with an estimated glomerular filtration rate (eGFR) above 90 mL/min/1.73 m². Haemoglobin is 141 g/L, within the normal range (typically 130-180 g/L for males and 115-165 g/L for females), and platelet count is 429×10⁹/L, which lies at the upper end of the normal range (150-450×10⁹/L). There is no previous history of thrombosis or bleeding. He was on oral carbamazepine for seizure control. The patient underwent right hip hemiarthroplasty, which was uncomplicated. Post-operative thromboprophylaxis was initially planned with low-molecular-weight heparin (enoxaparin). However, because of his significant needle phobia, attempts to administer injectable medication caused extreme distress and agitation, posing a risk to both patient and staff. Hence, enoxaparin was considered unsuitable. The multidisciplinary team then discussed alternative oral anticoagulants in the patient's best interest. Vitamin K antagonists like warfarin were avoided due to concerns regarding poor compliance, as regular international normalised ratio (INR) monitoring would again pose challenges in view of his needle phobia. Rivaroxaban, commonly used after hip surgery, was also deemed unsuitable due to its major drug interaction with carbamazepine, a strong inducer of liver enzymes that significantly reduces plasma concentrations and the efficacy of rivaroxaban and apixaban. The patient was started on edoxaban 60 mg once daily. Blood levels of carbamazepine and edoxaban were monitored during treatment. The patient's serum carbamazepine level was found to be 4.8 milligrams per litre (mg/L), which falls within the lower end of the therapeutic range. The first sample was obtained before the morning dose of edoxaban to measure trough levels, and peak levels were assessed four hours after administration. For edoxaban, both peak and trough levels were assessed using standardised anti-factor Xa assays measuring 67.98 nanograms per millilitre (ng/mL) and 19 ng/mL, respectively. Due to the patient's poor cooperation, the venous blood sample was taken approximately four hours after the administration of lorazepam. Laboratory findings are summarised in Table 1.

**Table 1:** Summary of drug monitoring results **Reference ranges for peak and trough levels of edoxaban are approximate and may vary based on clinical guidelines and patient-specific factors. mg/L: milligrams per litre; ng/mL: nanograms per millilitre

Test	Measured value	Reference range	Units
Carbamazepine (total)	4.8	4-12	mg/L
Edoxaban (peak level)	67.98	60-200**	ng/mL
Edoxaban (trough level)	19	10-50**	ng/mL

After completing five weeks of edoxaban, as per the standard protocol for post-hip surgery thromboprophylaxis, the patient was discharged. No thromboembolic events or major bleeding was reported during the hospital stay or follow-up period. 

## Discussion

Managing anticoagulation in patients with multiple medical and behavioural challenges requires a highly individualised and collaborative approach. In routine cases following hip fracture surgery, low-molecular-weight heparin or DOACs are commonly used. However, this case involved several unique barriers that made standard protocols unsuitable, highlighting the need for flexibility and clinical judgement.

Rivaroxaban and apixaban, two DOACs frequently used in orthopaedic settings, were considered but ultimately avoided due to known drug interactions with carbamazepine. As a strong inducer of CYP3A4 and P-glycoprotein, carbamazepine can substantially reduce the blood levels of these agents, potentially leading to treatment failure. A pharmacokinetic modelling study by Ngo et al. supports this concern, showing the accelerated clearance of rivaroxaban in the presence of carbamazepine [5]. 

Emerging data suggest that edoxaban may be less affected by enzyme induction. Research by Lenard et al. involving healthy volunteers found that carbamazepine had a smaller impact on edoxaban concentrations compared to other factor Xa inhibitors (FXaI) [6]. This pharmacokinetic profile made edoxaban a reasonable option in our patient, especially given the limited alternatives. 

To support this decision, drug levels were measured, showing peak and trough concentrations of edoxaban within acceptable ranges. These findings, along with the absence of any thromboembolic or bleeding events, suggest that edoxaban may be a safer choice in similar cases. While routine monitoring of DOAC levels is not standard practice, selective measurement in complex patients, like this one, can help guide therapy and ensure effectiveness [7]. 

This case also reflects a broader issue in elderly or institutionalised populations, where polypharmacy, frailty, and limited social support often intersect. Managing DOAC therapy in these patients can be difficult, particularly when other high-risk medications are involved. A review by Galgani et al. highlighted the lack of definitive guidance in such scenarios, while Mar et al. emphasised how drug interactions can significantly alter anticoagulant activity [3,8]. 

Interestingly, retrospective evidence from cohort analyses indicated that increased FXaI exposure (like edoxaban) is associated with major bleeding events, as well as an increased risk of stroke and all-cause mortality [9-18]. 

Ultimately, this case adds to a small but growing body of evidence that supports the cautious use of edoxaban in patients taking enzyme-inducing antiepileptic drugs. It also underscores the importance of multidisciplinary collaboration with pharmacy and the potential value of laboratory support in tailoring anticoagulation strategies when standard options are not appropriate. 

## Conclusions

While there is limited published evidence on the use of edoxaban in patients receiving enzyme-inducing antiepileptic drugs, this case provides practical insight in an area where guidance is lacking. It supports the consideration of edoxaban in highly selected, high-risk patients for whom standard injectable or monitoring-intensive therapies are unsuitable. However, conclusions cannot be generalised from a single case, and the timing of drug level monitoring limits interpretation. Further data are required to clarify the safety, efficacy, and pharmacokinetic reliability of edoxaban in this context. This case shows the importance of a personalised, evidence-based approach, with DOAC monitoring used to guide decisions in complex patients who do not fit standard treatment options.
